# Experience-dependent modulation of prosocial touch in mice

**DOI:** 10.1016/j.isci.2026.115102

**Published:** 2026-02-20

**Authors:** Yuhan Sun, Weizhe Hong, Ye Emily Wu

**Affiliations:** 1 Department of Neurobiology, David Geffen School of Medicine, University of California, Los Angeles, Los Angeles, CA, USA; 2 Department of Biological Chemistry, David Geffen School of Medicine, University of California, Los Angeles, Los Angeles, CA, USA; 3 Department of Bioengineering, Samueli School of Engineering, University of California, Los Angeles, Los Angeles, CA, USA

**Keywords:** neuroscience, behavioral neuroscience, cognitive neuroscience, psychology

## Abstract

Prosocial behaviors that benefit others are vital for social cohesion and collective well-being. How prior experiences shape future prosocial actions remains a critical open question. Mice can respond to distressed social partners with prosocial touch (allogrooming), alleviating stress in recipients. We show that mice that previously received prosocial contact following stress subsequently exhibited enhanced allogrooming toward stressed partners, and the amount of allogrooming previously received predicted the amount later displayed toward others. Notably, attenuation of peripheral tactile sensation during the receipt of prosocial contact diminished its impact on future allogrooming behavior, suggesting a critical role of somatosensation in mediating this phenomenon. Furthermore, receipt of prosocial contact was associated with increased neuronal activation in the posterior intralaminar thalamic nucleus, a region implicated in processing social touch information and regulating allogrooming behavior. Collectively, our findings offer insights into experience-dependent modulation of prosocial touch and its underlying sensory and neural mechanisms.

## Highlights

• Receipt of prosocial contact enhances allogrooming toward stressed partners in mice
• Previously received allogrooming predicts allogrooming toward stressed partners
• Experience-dependent modulation of allogrooming may require somatosensation
• Receipt of prosocial contact leads to increased neural activity in the PIL

## Graphical abstract

**Figure:**
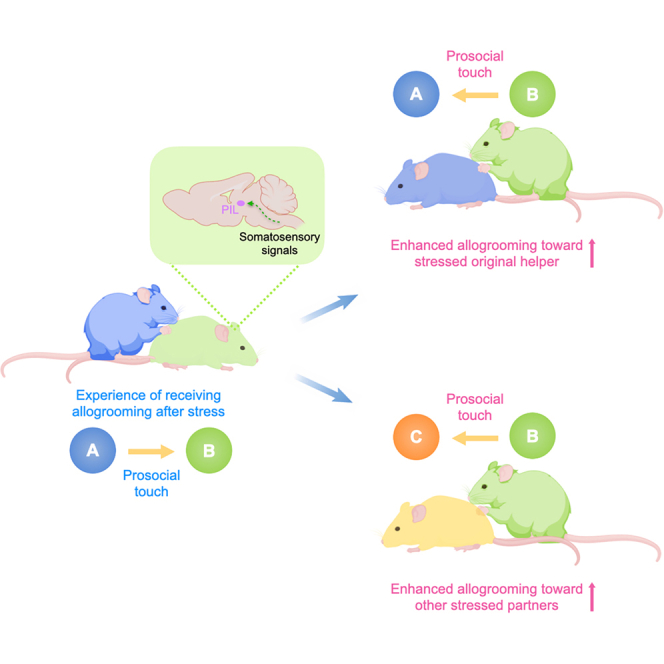

## Introduction

The ability to perceive others’ needs or distress and take prosocial actions that improve their conditions plays a critical role in fostering social cohesion and enhancing collective well-being in social species, including humans.^1^^,^^2^^,^^3^^,^^4^ Prosocial behaviors have been documented across many species, and recent studies have established several models of emotional contagion and prosocial interactions in rodents.^1^^,^^2^^,^^3^^,^^5^^,^^6^^,^^7^^,^^8^^,^^9^^,^^10^^,^^11^^,^^12^^,^^13^^,^^14^^,^^15^^,^^16^ For instance, in response to conspecifics experiencing emotional stress or physical injuries, laboratory mice can exhibit affiliative allogrooming targeted at various body parts or allolicking directed at the injury site, which can help alleviate the recipients’ distress or pain.^7^^,^^10^ Although research to date has largely examined these behaviors in mice in one-time, unidirectional interactions, long-term prosocial relationships often rely on repeated, bidirectional exchanges, and can involve multiple individuals. However, how prior experiences may influence future prosocial behaviors in repeated interactions remains incompletely understood.

Various types of prior experiences can modulate future prosocial decisions. For instance, receiving help from others can increase an individual’s propensity to provide aid to the original helper or other individuals.^17^^,^^18^^,^^19^^,^^20^ This reciprocal process can facilitate stable cooperation and has been proposed as a key mechanism in the evolution of prosocial behavior.^17^^,^^18^^,^^19^^,^^20^ Reciprocity is widespread among humans and has also been observed in other species.^17^^,^^18^^,^^19^^,^^20^^,^^21^^,^^22^^,^^23^^,^^24^ Recent studies have shown that rats are more likely to provide food to others or allogroom others for hygiene if they have previously received such prosocial actions.^25^^,^^26^^,^^27^^,^^28^^,^^29^^,^^30^ Additionally, prior experience of engaging in prosocial behavior can facilitate future prosocial actions in instrumental tasks in rats, suggesting a role of learning and reinforcement.^5^^,^^31^ However, how different types of prior experience may influence prosocial behavior toward distressed conspecifics in mice, and the underlying mechanisms of this modulation, are yet to be explored.

Here, we developed paradigms in mice to investigate how prior experiences modulate prosocial allogrooming behavior toward distressed conspecifics. We found that mice that previously received prosocial contact, including allogrooming, following stress subsequently exhibited elevated allogrooming toward stressed partners. Additionally, the amount of allogrooming previously received predicted the level of allogrooming subsequently directed toward others. Meanwhile, prior experience of engaging in prosocial contact did not significantly alter allogrooming behavior during subsequent interactions. Notably, attenuating peripheral somatosensation during the receipt of prosocial contact diminished its effect on future allogrooming behavior, suggesting a critical role of physical touch in mediating this phenomenon. Furthermore, the posterior intralaminar thalamic nucleus (PIL), a brain region implicated in processing social touch signals and regulating allogrooming,^32^^,^^33^^,^^34^ showed increased neuronal activation following the receipt of prosocial contact. Together, our findings demonstrate experience-dependent modulation of prosocial allogrooming behavior in mice and provide insight into the underlying sensory and neural mechanisms.

## Results

### Prior experience of receiving prosocial contact enhances future allogrooming behavior toward another stressed partner

**Figure 1:**
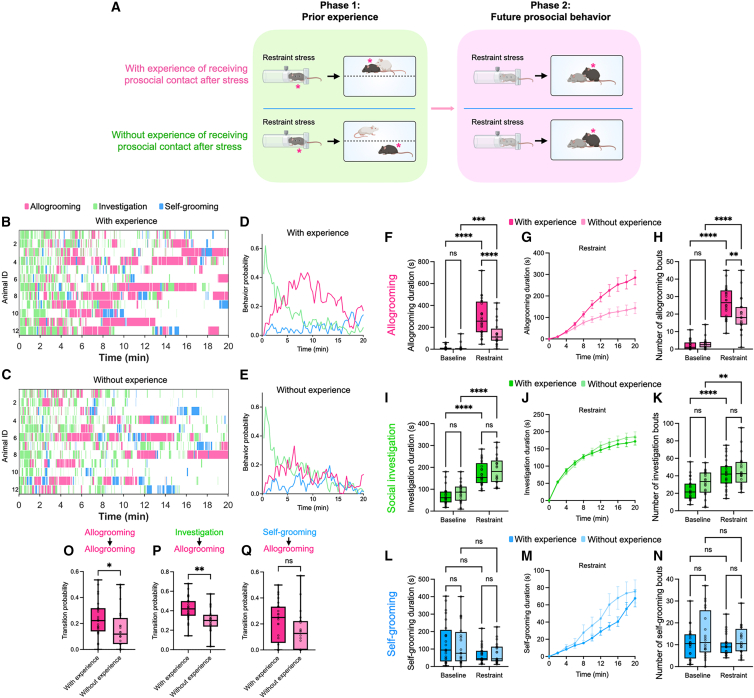
Prior receipt of prosocial contact enhances future allogrooming behavior toward another partner (A) Schematic of the two-phase experimental paradigm for evaluating the effect of prior receipt of prosocial contact on future allogrooming behavior toward another stressed partner. In the first phase, subject mice were exposed to an acute stressor, after which they either received (the “with-experience” group) or did not receive (the “without-experience” group) direct prosocial contact from a partner. In the second phase, the subjects’ allogrooming and other behaviors during subsequent interaction with a second, stressed partner were examined. Subject animals are indicated by asterisks. All mice were of the C57BL/6J strain, with different coat colors used in the schematic to distinguish between individuals. (B and C) Example raster plots showing allogrooming, social investigation, and self-grooming behaviors exhibited by subject animals during interactions with stressed partners in the second phase of the experiment in the “with-experience” (B) and “without-experience” (C) groups. Each row represents an individual subject animal. (D and E) The probability of subject animals displaying allogrooming, social investigation, and self-grooming behaviors at different time points during interactions with stressed partners in the “with-experience” (D) and “without-experience” (E) groups. Data at each time point represent the mean value across all animals. (F–N) The total duration (F, I, and L), cumulative duration (mean ± SEM) at various time points (G, J, and M), and total number of episodes (H, K, and N) of allogrooming (F–H), social investigation (I–K), and self-grooming (L–N) displayed by subject animals during interactions with unstressed or stressed partners in the “with-experience” and “without-experience” groups. (O–Q) Transition probabilities between different types of behaviors displayed by subject mice during interactions with stressed partners in the “with-experience” and “without-experience” groups. (O) Probability of transitioning from an episode of allogrooming to a subsequent episode of allogrooming. (P) Probability of transitioning from an episode of social investigation to a subsequent episode of allogrooming. (Q) Probability of transitioning from an episode of self-grooming to a subsequent episode of allogrooming. *n* = 24 male mice in the “with-experience” group and 20 male mice in the “without-experience” group. The whiskers in the boxplots indicate the range from the minimum to the maximum value. (F, H, I, K, L, and N) Two-way repeated measures analysis of variance (ANOVA) with post hoc Šidák multiple comparisons test. (O–Q) Two-sided Wilcoxon rank-sum test. ∗∗∗∗*p* < 0.0001. ∗∗∗*p* < 0.001. ∗∗*p* < 0.01. ∗*p* < 0.05. ns, not significant. See Table S1 for additional statistical details. See also Figures S1 and S2.

To investigate how prior receipt of prosocial contact influences future prosocial behavior in mice, we developed a two-phase paradigm (Figure 1A; STAR Methods). In the first phase, a male subject mouse was acutely stressed via physical restraint and subsequently reunited with a male social partner. In the “with-experience” group, the animals interacted freely, allowing the partner to engage in close physical contact with the subject, including allogrooming and other forms of social touch (Figure S1A). In the “without-experience” group, the animals were separated by a wire mesh divider, which permitted the transmission of olfactory, visual, and auditory cues but prevented direct physical contact. Following this initial interaction, the subject was co-housed with a second, naive social partner. In the second phase, this partner was exposed to acute restraint stress and then reintroduced to the subject, allowing us to examine the subject’s allogrooming and other behaviors during interaction with the stressed partner.

We found that subjects that previously received prosocial contact from a partner following stress exposure exhibited significantly higher levels of allogrooming during subsequent interactions with another stressed social partner than those that did not receive prior prosocial contact (Figures 1B–1H). While both groups displayed increased allogrooming toward stressed partners compared to unstressed partners (Figures 1F and 1H), the “with-experience” group exhibited significantly greater total duration (Figures 1B, 1C, 1F, and 1G) and number of episodes (Figure 1H) of allogrooming toward stressed partners than the “without-experience” group. Meanwhile, the level of baseline allogrooming toward unstressed partners did not differ between the two groups (Figures 1F and 1H). Analysis of the temporal dynamics of allogrooming revealed that this behavior was initiated shortly upon encountering stressed partners, peaking between 5 and 10 min in both groups (Figures 1D and 1E). The enhancement of allogrooming in the “with-experience” group was similarly observed 24 h or 48 h after the initial experience (Figure S1B), and appeared to be independent of the dominance relationship between the subject animal and the partner used in phase 2 (Figure S1C).

To investigate whether the observed effect was specific to allogrooming, we also examined other behaviors. We found that social sniffing toward stressed partners, an investigatory behavior for sensory sampling, showed no significant difference between the two groups (Figures 1B–1E and 1I–1K), suggesting similar levels of general investigatory drive. Additionally, the subjects’ self-directed grooming behavior remained unchanged (Figures 1B–1E and 1L–1N), suggesting no general increase in grooming behavior. We also characterized huddling behavior, another affiliative behavior typically performed for warmth or safety, and observed no significant difference between the two groups (Figures S1D and S1E). We further examined the transition probabilities between different behaviors and observed a significant increase in the probability of subject animals transitioning from one episode of allogrooming or social investigation to another episode of allogrooming toward stressed partners (Figures 1O–1Q) in the “with-experience” group compared to the “without-experience” group, consistent with an increased frequency of allogrooming behavior in the former group.

We next examined whether female mice exhibit similar reciprocity using the same paradigm. Previous work showed that while female mice also exhibit allogrooming toward stressed female partners, the overall level of allogrooming is substantially lower than that observed in male pairs.^7^ Consistent with this finding, we observed that stressed female subjects received significantly less allogrooming from their female partners during phase 1 of the paradigm than male subjects (Figure S2A). Moreover, the level of allogrooming, social investigation, and self-grooming displayed by female subjects in phase 2 did not significantly differ between the “with-experience” and “without-experience” groups (Figures S2B–S2G).

### Prior experience of receiving prosocial contact enhances future allogrooming behavior toward the original helper

**Figure 2:**
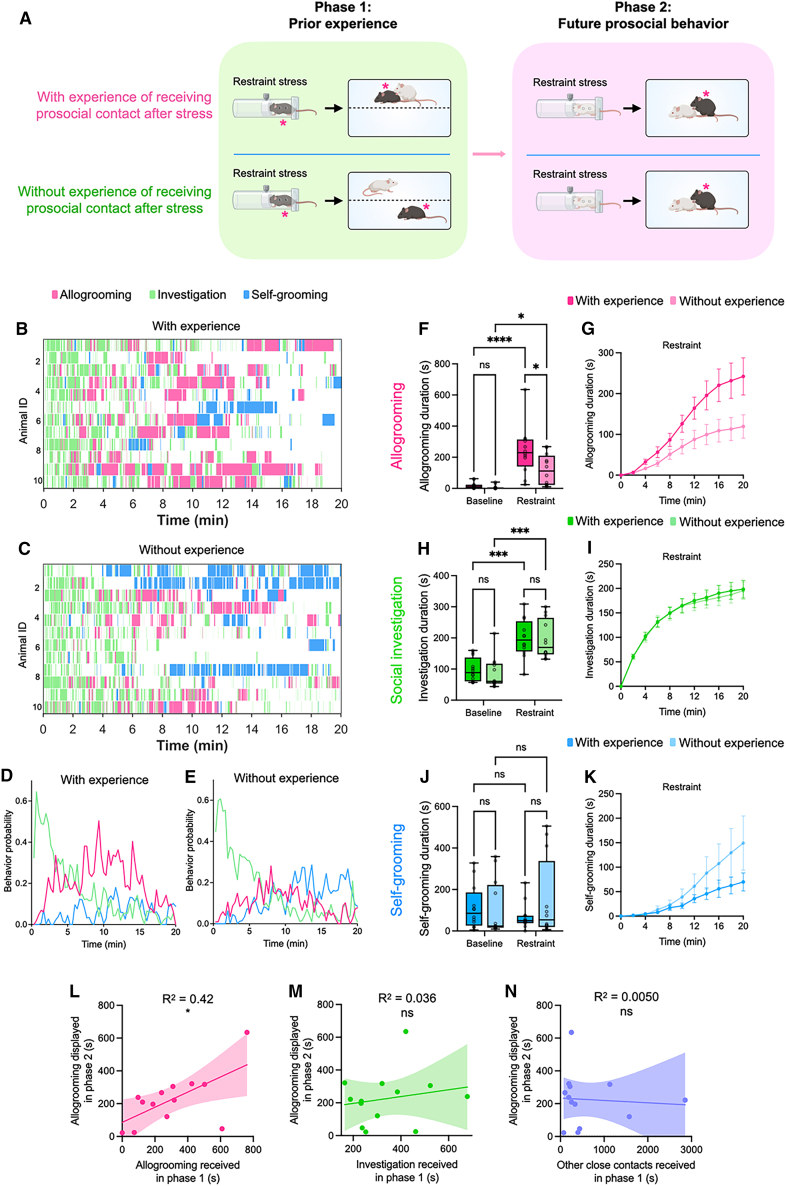
Prior receipt of prosocial contact enhances future allogrooming behavior toward the original helper (A) Schematic of the two-phase experimental paradigm for evaluating the effect of prior receipt of prosocial contact on future allogrooming behavior toward the original helper. In the first phase, subject mice were exposed to an acute stressor, after which they either received (the “with-experience” group) or did not receive (the “without-experience” group) direct prosocial contact from a partner. In the second phase, the same partner was exposed to acute stress, and the subjects’ allogrooming and other behaviors during interaction with this stressed partner were examined. Subject animals are indicated by asterisks. All mice were of the C57BL/6J strain, with different coat colors used in the schematic to distinguish between individuals. (B and C) Example raster plots showing allogrooming, social investigation, and self-grooming behaviors exhibited by subject animals during interactions with stressed partners in the second phase of the experiment in the “with-experience” (B) and “without-experience” (C) groups. Each row represents an individual subject animal. (D and E) The probability of subject animals displaying allogrooming, social investigation, and self-grooming behaviors at different time points during interactions with stressed partners in the “with-experience” (D) and “without-experience” (E) groups. Data at each time point represent the mean value across all animals. (F–K) The total duration (F, H, and J) and cumulative duration (mean ± SEM) at various time points (G, I, and K) of allogrooming (F and G), social investigation (H and I), and self-grooming (J and K) displayed by subject animals during interactions with unstressed or stressed partners in the “with-experience” and “without-experience” groups. (L–N) Correlations between the total duration of allogrooming displayed by subject animals in the “with-experience” group during phase 2 and different types of social contact received by the subject animals during phase 1. (L) Correlation with the total duration of allogrooming received in phase 1. (M) Correlation with the total duration of social investigation received in phase 1. (N) Correlation with the total duration of other forms of close contact in phase 1, including those occurring during huddling or locomotion. The linear regression lines (solid lines) and 95% confidence intervals (shaded areas) are shown. *n* = 12 male mice in the “with-experience” group and 12 male mice in the “without-experience” group in (D–K). *n* = 13 male mice in (L–N). One animal in the “with-experience” group that received 0 s of allogrooming was not included in the quantification in (D–K) but was included in the correlation analysis in (L–N). The whiskers in the boxplots indicate the range from the minimum to the maximum value. (F, H, and J) Two-way repeated measures analysis of variance (ANOVA) with post hoc Šidák multiple comparisons test. (L–N) Pearson correlation. ∗∗∗∗*p* < 0.0001. ∗∗∗*p* < 0.001. ∗∗*p* < 0.01. ∗*p* < 0.05. ns, not significant. See Table S1 for additional statistical details. See also Figures S1 and S2.

We next examined whether prior receipt of prosocial contact enhances allogrooming toward the original helper during subsequent interactions. In this paradigm, the partner that interacted with the subject in phase 1 was later exposed to acute stress in phase 2 and then reintroduced to the subject for interaction (Figure 2A). We found that subject animals that previously received prosocial contact exhibited increased allogrooming (Figures 2B–2G), but not social investigation or self-grooming (Figures 2H–2K), toward their original helpers when the helpers were stressed, compared to subjects without such experience. Additionally, we found that the amount of allogrooming toward the stressed original helper and a stressed second partner did not differ significantly (two-sided Wilcoxon rank-sum test, *p* = 0.46; Figures 1F and 2F), suggesting that this effect was not dependent on the partner’s identity.

To further assess the functional significance of previously received allogrooming in modulating future allogrooming behavior toward others, we examined the correlation between the amount of allogrooming that a stressed subject received from a helper in phase 1 and the amount of allogrooming the subject directed toward the stressed helper in phase 2. Notably, these measures were significantly positively correlated (Figure 2L). In contrast, the amount of social investigation or other forms of close contact (including those during huddling or locomotion) received by the subject in phase 1 did not correlate significantly with their allogrooming toward others in phase 2 (Figures 2M and 2N). These results indicate that previously received allogrooming, rather than other forms of close social interaction, is a primary predictor of future allogrooming toward others.

### The effect of prior prosocial contact is not due to lasting changes in stress levels

**Figure 3:**
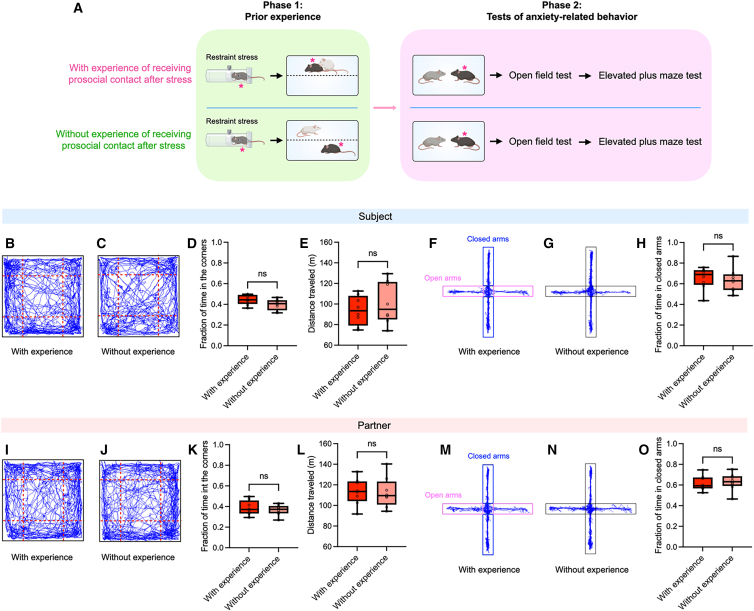
The effect of receiving prosocial contact on subsequent allogrooming does not result from changes in general stress levels (A) Schematic of the two-phase experimental paradigm for evaluating the effect of prior receipt of prosocial contact on baseline stress levels. Both the subjects (indicated by asterisks) and their co-housed partners were tested. All mice were of the C57BL/6J strain, with different coat colors used in the schematic to distinguish between individuals. (B, C, I, and J) Example locomotion trajectories in the open field for subject mice (B and C) and their co-housed partners (I and J) in the “with-experience” (B and I) and “without-experience” groups (C and J). (D, E, K and L) Fraction of time spent in the corners (D and K) and total distance traveled (E and L) in the open field for subject mice (D and E) and their partners (K and L) in the “with-experience” and “without-experience” groups. (F, G, M, and N) Example locomotion trajectories in the elevated plus maze for subject mice (F and G) and their co-housed partners (M and N) in the “with-experience” (F and M) and “without-experience” groups (G and N). (H and O) Fraction of time spent in the closed arms of the elevated plus maze for subject mice (H) and their partners (O) in the “with-experience” and “without-experience” groups. *n* = 8 male subjects and 8 male partners for both the “with-experience” and “without-experience” groups. The whiskers in the boxplots indicate the range from the minimum to the maximum value. (D, E, H, K, L, and O), Two-sided Wilcoxon rank-sum test. ns, not significant. See Table S1 for additional statistical details.

Previous studies have shown that stressed mice that received prosocial contact from partners showed an acute reduction in stress levels compared to those without such experience.^6^^,^^7^^,^^35^ This raises the possibility that prior receipt of allogrooming in phase 1 of our current paradigm could produce a long-lasting effect on subjects’ stress levels, contributing to the behavioral differences observed in phase 2. To examine this, we conducted open-field and elevated plus maze tests ∼24 h after phase 1 to assess baseline stress levels in male subject mice that either received or did not receive allogrooming in phase 1 (Figure 3A). We found that subjects in the “with-experience” group and the “without-experience” group spent similar fractions of time in the corners of the open field, suggesting comparable levels of anxiety-related behavior (Figures 3B–3D). The total distance traveled also did not differ between the two groups, indicating similar locomotor activity (Figure 3E). Similarly, subjects in both groups spent a comparable fraction of time in the closed arms of the elevated plus maze, consistent with comparable anxiety levels (Figures 3F–3H). We also confirmed that the partners, which were co-housed with the subjects after phase 1, showed comparable levels of anxiety-related behavior in the open-field and elevated plus maze tests (Figures 3I–3O). These results suggest that mice in both the with- and without-experience groups had similarly recovered from the initial stress state by phase 2 and that the observed difference in allogrooming behavior between the two groups is unlikely to be attributable to differences in general stress levels at that time.

### The effect of prior prosocial contact is specific to the recipient but not the actor

**Figure 4:**
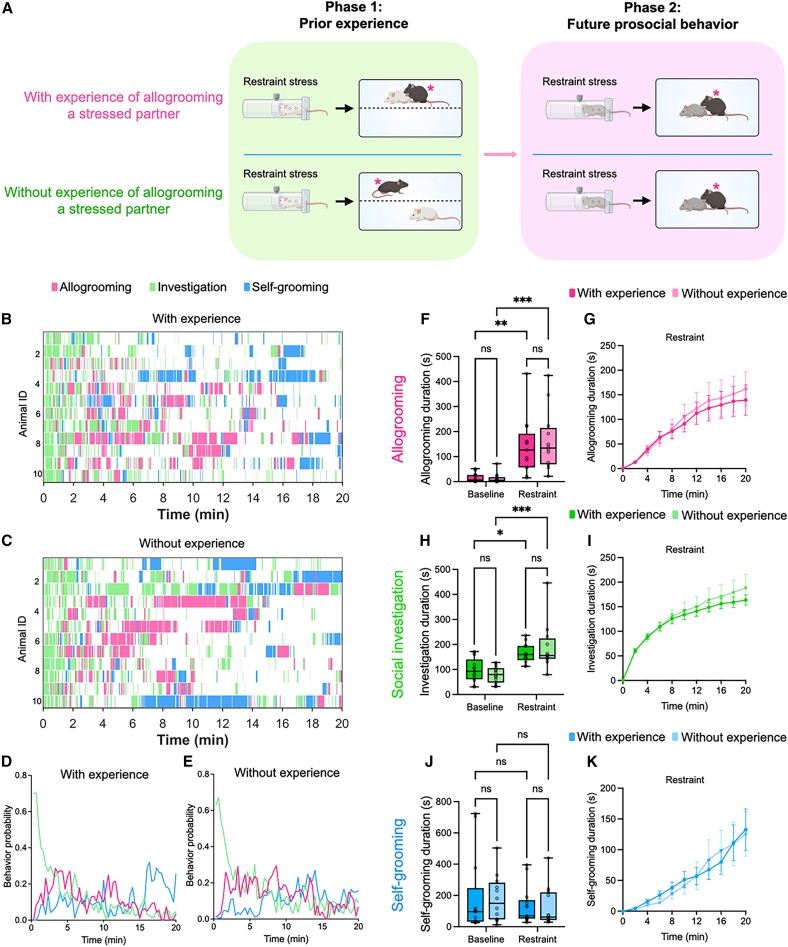
The effect of prior prosocial contact is observed in the recipient but not the actor (A) Schematic of the two-phase experimental paradigm for evaluating the effect of prior engagement in prosocial contact on future allogrooming behavior toward stressed partners. In the first phase, subject mice either engaged in direct prosocial contact toward a stressed partner (the “with-experience” group) or interacted with a stressed partner through a wire mesh divider to prevent close contact (the “without-experience” group). In the second phase, the subjects’ allogrooming and other behaviors during subsequent interactions with an unstressed or stressed partner were examined. Subject animals are indicated by asterisks. All mice were of the C57BL/6J strain, with different coat colors used in the schematic to distinguish between individuals. (B and C) Example raster plots showing allogrooming, social investigation, and self-grooming behaviors exhibited by subject animals during interactions with stressed partners in the second phase of the experiment in the “with-experience” (B) and “without-experience” (C) groups. Each row represents an individual subject animal. (D and E) The probability of subject animals displaying allogrooming, social investigation, and self-grooming behaviors at different time points during interactions with stressed partners in the “with-experience” (D) and “without-experience” (E) groups. Data at each time point represent the mean value across all animals. (F–K) The total duration (F, H, and J) and cumulative duration (mean ± SEM) at various time points (G, I, and K) of allogrooming (F and G), social investigation (H and I), and self-grooming (J and K) displayed by subject animals during interactions with unstressed or stressed partners in the “with-experience” and “without-experience” groups. *n* = 13 male mice in the “with-experience” group and 12 male mice in the “without-experience” group. The whiskers in the boxplots indicate the range from the minimum to the maximum value. (F, H, and J), Two-way repeated measures analysis of variance (ANOVA) with post hoc Šidák multiple comparisons test. ∗∗∗*p* < 0.001. ∗∗*p* < 0.01. ∗*p* < 0.05. ns, not significant. See Table S1 for additional statistical details.

Given that recipients of prosocial contact showed increased allogrooming in future interactions, we next examined whether the actors that previously provided prosocial contact also showed enhanced allogrooming in subsequent interactions. Prior research using instrumental tasks in rats has shown that repeated interactions improved prosocial performance, suggesting a role for learning and reinforcement.^5^^,^^31^ To investigate this in the context of allogrooming, male subject mice first interacted with a stressed partner, either freely (the “with-experience” group) or through a wire mesh divider (the “without-experience” group). Subsequently, we examined their behaviors during free interactions with a second partner in a neutral or stressed state (Figure 4A). We observed no significant differences in allogrooming, social investigation, or self-grooming behavior between the “with-experience” and the “without-experience” groups during interactions with either neutral or stressed partners (Figures 4B–4K). This suggests that previous prosocial contact does not enhance future allogrooming behavior of the actor, in line with the idea that allogrooming toward stressed partners is a largely innate behavior that does not require learning.

### Attenuation of somatosensation diminishes the effect of receiving prosocial contact on future allogrooming behavior

**Figure 5:**
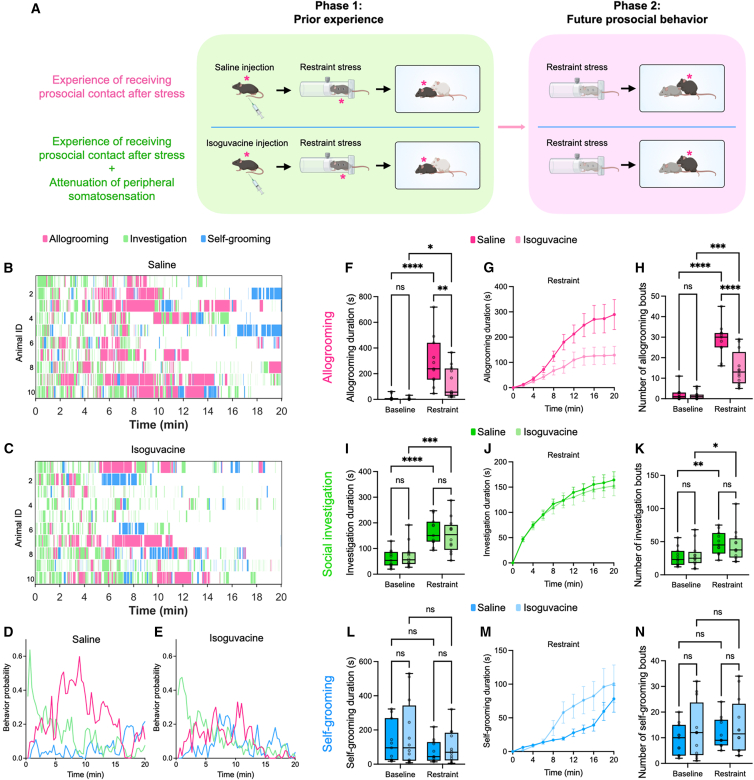
Attenuation of peripheral somatosensation diminishes the effect of prior prosocial contact (A) Schematic of the two-phase experimental paradigm for evaluating how attenuating peripheral somatosensation influences the effect of prior receipt of prosocial contact on subsequent allogrooming toward stressed partners. In the first phase, subject mice were intraperitoneally injected with either isoguvacine (the “isoguvacine” group) or saline (the “saline” group, control) before being exposed to an acute stressor and subsequently receiving prosocial contact from a partner. In the second phase, the subjects’ allogrooming and other behaviors during subsequent interactions with a second, stressed partner were examined. Subject animals are indicated by asterisks. All mice were of the C57BL/6J strain, with different coat colors used in the schematic to distinguish between individuals. (B and C) Example raster plots showing allogrooming, social investigation, and self-grooming behaviors exhibited by subject animals during interactions with stressed partners in the second phase of the experiment in the “saline” (B) and “isoguvacine” (C) groups. Each row represents an individual subject animal. (D and E) The probability of subject animals displaying allogrooming, social investigation, and self-grooming behaviors at different time points during interactions with stressed partners in the “saline” (D) and “isoguvacine” (E) groups. Data at each time point represent the mean value across all animals. (F–N) The total duration (F, I, and L), cumulative duration (mean ± SEM) at various time points (G, J, and M), and total number of episodes (H, K, and N) of allogrooming (F–H), social investigation (I–K), and self-grooming (L–N) displayed by subject animals in the “isoguvacine” and “saline” groups during interactions with unstressed or stressed partners. *n* = 12 male mice in the “isoguvacine” group and 11 male mice in the “saline” group. The whiskers in the boxplots indicate the range from the minimum to the maximum value. (F, H, I, K, L, and N) Two-way repeated measures analysis of variance (ANOVA) with post hoc Šidák multiple comparisons test. ∗∗∗∗*p* < 0.0001. ∗∗∗*p* < 0.001. ∗∗*p* < 0.01. ∗*p* < 0.05. ns, not significant. See Table S1 for additional statistical details. See also Figures S3 and S4.

We next investigated what sensory cues may be crucial for mediating the effect of prior prosocial experience. Given that allogrooming is a form of gentle touch that can generate a comforting effect through specialized mechanosensory pathways,^36^^,^^37^^,^^38^^,^^39^ we hypothesized that somatosensation is a necessary component. Alternatively, other sensory inputs (such as olfactory, visual, and auditory cues) during prosocial contact might be sufficient for mediating this effect. To directly test this, we acutely attenuated tactile sensitivity in stressed male subject mice while they freely interacted with partners, by injecting the subjects with isoguvacine, a peripherally restricted GABA_A_ receptor agonist that acts directly on mechanosensory neurons^40^^,^^41^^,^^42^^,^^43^ (Figure 5A). Application of this drug has been shown to reduce somatosensory activity and alter social touch-related responses in mice.^40^^,^^41^ Subjects that received saline injection served as controls (Figure 5A). Notably, isoguvacine administration in subjects during previous prosocial contact significantly reduced both the total duration and frequency of subsequent allogrooming toward stressed partners (Figures 5B–5H). Meanwhile, social investigation and self-grooming behaviors were not significantly changed (Figures 5B–5E and 5I–5N).

To determine whether this effect was due to a difference in the amount of prior social contact received by saline- and isoguvacine-injected subjects, we compared the duration of allogrooming and social investigation they received in the first phase of the experiment and observed no significant differences between the two groups (Figures S3A–S3C). Additionally, to examine if isoguvacine administration alone, without prosocial experience, influences future allogrooming behavior, we injected either isoguvacine or saline into subject animals that did not experience stress or prosocial interaction in the first phase (Figure S4A). We observed no significant differences in allogrooming, social investigation, or self-grooming between the two groups in the second phase (Figures S4B–S4D), indicating that the effect of isoguvacine was dependent on prior prosocial interaction. Together, these results support a critical role of somatosensation in the modulation of future allogrooming behavior by prior prosocial contact.

### Receipt of prosocial contact is associated with increased neuronal activation in the PIL

**Figure 6:**
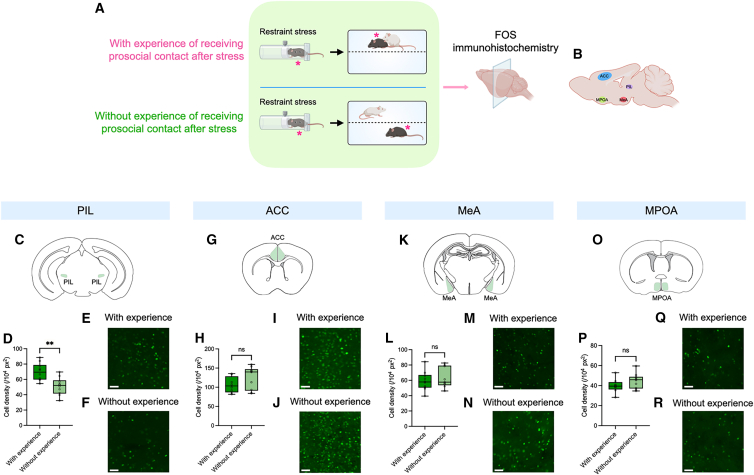
Receipt of prosocial contact increases neuronal activation in the PIL (A) Schematic of the experimental paradigm for examining Fos expression following the receipt of prosocial contact. Subject mice (indicated by asterisks) were exposed to an acute stressor, after which they either received (the “with-experience” group) or did not receive (the “without-experience” group) direct prosocial contact from a partner. Subject mice were sacrificed after 2.5 h of interaction, and their brains were sectioned and immunostained for Fos protein. (B) Schematic showing the different brain areas examined. (C, G, K, and O) Schematic of the PIL (C), ACC (G), MeA (K), and MPOA (O) in coronal sections. (D, H, L, and P) Quantification of the density of Fos-positive cells in the PIL (D), ACC (H), MeA (L), and MPOA (P) in subject animals in the “with-experience” and “without-experience” groups. (E, F, I, J, M, N, Q, and R) Example fluorescence images showing Fos-positive cells in the PIL (E and F), ACC (I and J), MeA (M and N), and MPOA (Q and R) in subject animals in the “with-experience” (E, I, M, and Q) and “without-experience” (F, J, N, and R) groups. *n* = 10 hemispheres (from 5 male mice) in the “with-experience” group and 10 hemispheres (from 5 male mice) in the “without-experience” group in (D). *n* = 8 hemispheres (from 4 male mice) in the “with-experience” group and 10 hemispheres (from 5 male mice) in the “without-experience” group in (H). *n* = 10 hemispheres (from 5 male mice) in the “with-experience” group and 9 hemispheres (from 5 male mice) in the “without-experience” group in (L). *n* = 8 hemispheres (from 4 male mice) in the “with-experience” group and 10 hemispheres (from 5 male mice) in the “without-experience” group in (P). The whiskers in the boxplots indicate the range from the minimum to the maximum value. (D, H, L, and P) Two-sided Wilcoxon rank-sum test. ∗∗*p* < 0.01. ns, not significant. Scale bars, 50 μm. See Table S1 for additional statistical details.

To explore the potential neural substrate mediating the effect of prior experience on future allogrooming behavior, we examined the expression of the neural activity marker Fos following the first phase of our behavioral paradigm, comparing stressed animals that either received or did not receive prosocial contact from their partners (Figures 6A and 6B). We considered the posterior intralaminar thalamic nucleus (PIL) as a candidate region, as it is involved in processing sensory information, including somatosensory signals, and has been shown to regulate allogrooming behavior in rats,^32^^,^^33^^,^^34^ raising the possibility that it may play a role in mediating the modulation of prosocial allogrooming by social touch. Notably, we found that stressed animals that received prosocial contact exhibited a significantly higher number of Fos-positive cells in the PIL than animals that did not (Figures 6C–6F). We also examined several other brain areas involved in regulating prosocial allogrooming behavior, including the anterior cingulate cortex (ACC), the medial amygdala (MeA), and the medial preoptic area (MPOA).^6^^,^^7^ In contrast to the PIL, no significant increases in Fos-positive cells were observed in the with-experience group in any of these regions (Figures 6G–6R). These findings suggest a potential role for the PIL in mediating the effect of receiving prosocial touch on future allogrooming behavior.

## Discussion

A fundamental question regarding prosociality is how behaviors that primarily benefit others could have evolved. One important mechanism proposed to underlie the evolution of prosociality is reciprocity, where prior receipt of help increases the propensity to aid the previous helper (direct reciprocity) and/or other individuals (generalized reciprocity),^17^^,^^18^^,^^19^^,^^20^ thereby offsetting the costs of helping others through future returns. This mechanism can facilitate stable cooperation among those who interact repeatedly in groups and allow prosocial interactions between two individuals to potentially influence a larger social group.^17^^,^^18^^,^^19^^,^^20^

Previous research has shown that rats exhibit increased prosocial behavior following the receipt of food provisioning or hygienic assistance through allogrooming.^25^^,^^26^^,^^27^^,^^28^^,^^29^^,^^30^ For instance, female rats that had been helped by others in food-provisioning tasks were more likely to assist both the original helpers and other individuals in subsequent tasks, with stronger helping behavior toward the original helpers.^26^^,^^44^ These findings suggest that female rats exhibit both generalized and direct reciprocity. In contrast, male rats increased helping behavior only toward partners that had previously assisted them, but not toward other individuals, suggesting that male rats exhibit direct, but not generalized reciprocity.^45^

In this study, we show that male mice with previous experience of receiving prosocial allogrooming after stress subsequently exhibited increased allogrooming toward other stressed partners, with comparable increases toward both the original helpers and other individuals. This provides the first experimental evidence for generalized reciprocity in prosocial allogrooming behavior in mice. The finding that both rats and mice exhibit generalized reciprocity, whereas only rats exhibit direct reciprocity, aligns with the notion that generalized reciprocity represents a more basic form of reciprocity, as it does not require recognition of specific partners or memory of previous encounters with them.^18^^,^^26^^,^^46^ Alternatively, this difference may reflect the use of distinct behavioral paradigms. In contrast, female mice received significantly less allogrooming from female partners than that observed in male pairs and subsequently exhibited no significant increases in allogrooming toward stressed partners. In light of our finding that the amount of previously received allogrooming positively correlated with the amount of allogrooming subsequently displayed toward others in male mice, the lack of reciprocity in females in our current paradigm might be partially attributable to the low level of allogrooming they received during prior experience. The mechanisms underlying these species and sex differences in prosocial reciprocity remain an important topic for future investigation.

The development of a relatively simple and fast paradigm for prosocial reciprocity in mice offers new opportunities for further investigation of its underlying neural mechanisms using diverse molecular and genetic tools in mice. The two-phase behavioral paradigm employed in the current study could also be adapted to characterize reciprocity in other forms of prosocial behavior in mice, such as helping behavior toward conspecifics in pain^10^ and aversion of harm to conspecifics.^47^ This paradigm may also be applied to examine potential reciprocation of different types of prosocial behaviors across contexts, as shown in previous research in rats.^28^

In this study, we examined prosocial reciprocity toward familiar cage-mate partners. Prosocial behavior often exhibits a familiarity bias, with individuals more likely to help familiar conspecifics than strangers.^1^^,^^48^ Indeed, previous research in prairie voles has demonstrated elevated prosocial allogrooming toward familiar partners compared to strangers.^6^ How social familiarity influences reciprocity in prosocial allogrooming awaits further investigation. Additionally, although we found that prior engagement in allogrooming was not sufficient to enhance future allogrooming by the actors under our experimental conditions, whether more robust or prolonged experience could yield a more pronounced effect remains to be determined.

We also investigated the potential mechanisms by which receiving prosocial contact increases future prosocial drive. We found that this effect was unlikely to be linked to long-term changes in the animals’ stress levels. Through pharmacological manipulation, we show that somatosensation during prior prosocial interactions likely plays a crucial role in this phenomenon. While previous research has indicated that olfactory cues mediate the impact of prosocial experiences on future behavior in an instrumental cooperative task in rats,^30^ our findings highlight that prior experiences can influence future interactions through distinct sensory modalities in different prosocial contexts and suggest gentle social touch as a potential means of enhancing positive social interactions. Whether other forms of gentle touch, including artificially delivered stimulation, can produce similar effects, and whether such effects require prior stress experience, remain important questions for future research. Finally, our finding of increased neuronal activation in the PIL—a brain region involved in both processing social touch and regulating allogrooming—following the initial experience suggests that it may link the receipt of prosocial contact to the modulation of future allogrooming, potentially through plasticity-based mechanisms. Collectively, our study demonstrates that prior experience modulates prosocial allogrooming in mice and identifies a critical sensory modality and a potential neural substrate underlying this process.

### Limitations of the study

Our findings lead to several questions that remain to be addressed. First, while we uncovered changes in neuronal activation in the PIL following the receipt of prosocial contact, the causal role of the PIL in mediating reciprocity in prosocial allogrooming remains to be determined through functional manipulation. Whether reciprocity in prosocial allogrooming involves select neuronal subpopulations and downstream circuits of the PIL also warrants further investigation. Second, our Fos staining experiments reveal cumulative changes in neuronal activation in the PIL after receiving prosocial contact. How single-neuron and ensemble activity dynamics are modulated specifically during the receipt of allogrooming, and how these dynamics lead to long-term changes that underlie the lasting effects of prior experience, will require examination using techniques with higher temporal and spatial resolution, such as *in vivo* calcium imaging. Additionally, although we observed an effect of prior experience on subsequent allogrooming at 24–48 h after the initial experience, whether this effect persists over longer time periods (such as days to weeks) remains to be determined. Moreover, whether prosocial reciprocity is present in other mouse strains and whether it varies across tasks or social contexts awaits systematic characterization. Furthermore, although we have focused on behavioral changes following prosocial experience in this study, additional physiological and biochemical measures, such as body temperature, respiration rate, heart rate, and hormone levels, would importantly complement our behavioral analyses in elucidating the effects of prior prosocial experience and represent an important direction for future research. Another limitation of this study is that our behavioral characterization was conducted through manual annotation by experimenters who were not blinded to the experimental groups. Although automated detection of complex social behaviors during close interactions, such as allogrooming, remains challenging, the future application of automated behavioral analysis tools, including deep learning-based methods, will enable more efficient and unbiased characterization of these complex interactions.

## Resource availability

### Lead contact

Further information and requests for resources and reagents should be directed to and will be fulfilled by the lead contact, Dr. Ye Emily Wu (ye.wu@ucla.edu).

### Materials availability

This study did not generate any new reagents or animal lines.

### Data and code availability

• All data generated or analyzed during this study are included in the article and supplemental information.
• Code for behavioral analysis (https://github.com/hongw-lab/Behavior_Annotator), animal pose tracking (https://github.com/talmolab/sleap/releases/tag/v1.3.3), and image analysis (https://qupath.github.io) is available on GitHub.
• Any additional information required to reanalyze the data reported in this paper is available from the lead contact upon request.

## Acknowledgments

We would like to thank Lauren Swanda, Mingmin Zhang, and Catherine Kim for technical assistance. Schematics in Figures 1, 2, 3, 4, 5, 6, S3, and S4 and the graphic abstract were in part created using BioRender (Wu, E. (2026) https://BioRender.com/54f0nxy). Schematics in Figures 6C, 6G, 6K, and 6O were modified from The Mouse Brain in Stereotaxic Coordinates.^49^ This work was supported in part by a Brain & Behavior Research Foundation Young Investigator Grant (to Y.E.W.) and a 10.13039/100000002National Institutes of Health grant (R01 MH130941 to W.H.).

## Author contributions

Y.E.W., Y.S., and W.H. designed the study; Y.S. carried out most of the experiments; Y.E.W. assisted with some experiments; Y.E.W. and Y.S. analyzed the data; Y.E.W. wrote the manuscript with inputs from W.H. and Y.S.; Y.E.W. supervised the project. All co-authors have read and approved the final version of the manuscript.

## Declaration of interests

The authors declare no competing interests.

## STAR★Methods

### Key resources table

**Table:** 

REAGENT or RESOURCE	SOURCE	IDENTIFIER
**Antibodies**		

Rabbit anti-Fos primary antibody	SYSY	Cat#: 226008; RRID: AB_2891278
Donkey anti-rabbit IgG Alexa Fluor 488 secondary antibody	ThermoFisher Scientific	Cat#: A-21206; RRID: AB_2535792

**Chemicals, peptides, and recombinant proteins**		

Isoguvacine	Sigma-Aldrich	Cat#: G002

**Experimental models: Organisms/strains**		

C57BL/6J mice	Jackson Laboratories	Strain#: 000664; RRID: IMSR_JAX:000664

**Software and algorithms**		

MATLAB	Mathworks (https://www.mathworks.com)	RRID: SCR_001622
GraphPad Prism	https://www.graphpad.com	RRID: SCR_002798
SLEAP	https://github.com/talmolab/sleap	RRID: SCR_021382
Behavior_Annotator	https://github.com/hongw-lab/Behavior_Annotator	N/A
QuPath	https://qupath.github.io	RRID: SCR_018257

### Experimental model and study participant details

#### Animals

Wild-type male and female C57BL/6J mice were purchased from Jackson Laboratories (strain #: 000664) at 8–12 weeks old and used for behavioral testing between ∼16–24 weeks old. Animals of matched age and sex were randomly assigned to experimental groups. Animals were housed in a 12-h light/dark cycle (21:00–9:00 light) at a temperature of 21–23°C with 30–70% humidity, with food and water *ad libitum*. All behavior experiments were performed during the dark cycle of the animals in a dark room illuminated by red light. All experimental procedures were carried out in compliance with the NIH Guide for Care and Use of Laboratory Animals and approved by the UCLA Institutional Animal Care and Use Committee (IACUC:2024-073).

### Method details

#### Behavior assays

Wild-type, same sex (male or female) C57BL/6J mice were co-housed for at least 4 weeks before behavioral testing. Animals were extensively habituated to handling procedures for at least 3 days prior to the behavioral test. Behaviors were recorded using a Point Grey Camera (FLIR) with a 30-Hz sampling rate and manually annotated frame-by-frame using a custom-written Python software (https://github.com/hongw-lab/Behavior_Annotator). Allogrooming was defined as visible licking and/or mouth contact accompanied by head bobbing movements directed at the body trunk, shoulders, and head of the partner mouse. Investigation was defined as the subject mouse orienting its snout toward the partner and positioning itself within half a head length of the partner. Self-grooming was defined as the subject mouse visibly licking its paws and grooming its face and/or body. Huddling was defined as two animals remaining in one location (typically the nest) with their bodies in contact.

##### Assessment of the effect of receiving prosocial contact on future allogrooming behavior

Same-sex mice were co-housed in groups of three (Figures 1 and S2) or in pairs (Figure 2) for at least 4 weeks before behavioral testing. One animal was randomly assigned as the “subject” and the others as the “partners”. The experiment comprised two phases. In the first phase, the subject mouse was removed from the home cage and subjected to 30 minutes of acute restraint stress. The subject was then reunited with a partner in the home cage, with a wire mesh divider placed in the middle of the cage during their interaction. In the “with-experience” group, the subject and the partner were placed on the same side of the divider, allowing the partner to engage in direct social contact, including allogrooming, toward the stressed subject. In the “without-experience” group, the two animals were placed on different sides of the divider, preventing direct physical contact while permitting olfactory, visual, and auditory communication. After one hour of interaction, the subject animal was co-housed with either a second partner (Figure 1) or the original helper (Figure 2) until the second phase. To prevent further direct physical contact between the animals during the interval between phase 1 and phase 2, a transparent, perforated Plexiglass divider was placed in the center of the cage, separating the two animals while permitting the exchange of visual, auditory, and olfactory cues. The second phase of the experiment was conducted ∼24–48 hours later. For the baseline session, the partner was removed from the home cage and placed into a separate cage with home cage bedding for 30 minutes. For the stress session, the partner was removed from the home cage and subjected to restraint stress for 30 minutes. After either separation only or stress, the partner was returned to the home cage to reunite with the subject, and the subject’s behaviors displayed during the first 20 minutes of the interaction were analyzed. The separation-only sessions were performed at least 3 hours before the stress sessions. In Figure 1, phase 2 was conducted at ∼24 hours after phase 1 in one subset of animals and after ∼48 hours in another subset. We observed a similar enhancement of allogrooming in the with-experience group at both 24 hours and 48 hours after the initial experience (Figure S1B), and there was no significant difference in the level of allogrooming in phase 2 between these two subsets (two-sided Wilcoxon rank-sum test, p = 0.47). This suggests that the effect of prior experience persists for at least 48 hours. These two cohorts of animals were therefore combined in Figure 1 to enhance statistical power. In Figure 2, phase 2 was conducted at ∼24 hours after phase 1 in all animals. To compute the probability of subject animals exhibiting various behaviors, we divided the interaction period into 20-second intervals. For each animal, we calculated the fraction of frames showing the behavior of interest in each interval and then averaged these fractions across all animals. To compute the transition probabilities between allogrooming, social investigation, and self-grooming (Figures 1O–1Q), we first merged episodes that were less than 1 second apart for each behavior. We then calculated the fraction of episodes of a given behavior that were immediately followed by each type of behavior (excluding periods during which none of the defined behaviors were observed).

##### Assessment of the effect of previous engagement in prosocial contact on future allogrooming behavior

Mice were co-housed in groups of three before behavioral testing. One animal was randomly assigned as the “subject” and the other two as the “partners”. The experiment consisted of two phases. In the first phase, a partner mouse was removed from the home cage and subjected to 30 minutes of acute restraint stress. The partner was then reunited with the subject mouse in the home cage, with a wire mesh divider placed in the middle of the cage during their interaction. In the “with-experience” group, the subject and the partner were placed on the same side of the divider, allowing the subject to engage in direct social contact, including allogrooming, toward the stressed partner. In the “without-experience” group, the two animals were placed on different sides of the divider, preventing direct physical contact while permitting olfactory, visual, and auditory communication. One hour later, the initial partner was removed, and a second partner was placed in the home cage and co-housed with the subject until the second phase. The second phase of the experiment was conducted ∼24 hours later as described in the “assessment of the effect of receiving prosocial contact on future allogrooming behavior” section.

##### Assessment of the effect of attenuating peripheral somatosensation

Mice were co-housed in groups of three before behavioral testing. One animal was randomly assigned as the “subject” and the other two as the “partners”. The subject was habituated to injection procedures by receiving daily 200 μL saline injections intraperitoneally for three days prior to the behavioral test. To evaluate how attenuation of somatosensation during prior prosocial interactions influences future prosocial behavior (Figures 5 and S3), the experiment consisted of two phases. In the first phase, the subject mouse was removed from the home cage and placed into a separate cage with home cage bedding. It was then injected intraperitoneally with either isoguvacine (Sigma-Aldrich, catalog # G002, at 20 mg/kg body weight) or 200 μL saline. 20 minutes post-injection, the subject mouse underwent 30 minutes of acute restraint stress before being reunited with a partner in the home cage. After one hour to allow the subject to recover from stress, the initial partner was removed, and a second partner was placed in the home cage and co-housed with the subject until the second phase. The second phase of the experiment was conducted ∼24 hours later as described in the “Assessment of the effect of receiving allogrooming on future allogrooming behavior” section.

To evaluate how isoguvacine administration alone, in the absence of prior prosocial experience, influences future prosocial behavior (Figure S4), the experiment comprised two phases. In the first phase, the subject mouse was removed from the home cage and injected intraperitoneally with either isoguvacine or 200 μL saline. It then remained in a separate cage with home cage bedding for 50 minutes before being reunited with a partner in the home cage. One hour later, the initial partner was removed, and a second partner was placed in the home cage and co-housed with the subject until the second phase. The second phase of the experiment was conducted ∼24 hours later, as described in the “assessment of the effect of receiving prosocial contact on future allogrooming behavior” section.

##### Open field test

The animals first underwent phase 1 of the experiment described in the “assessment of the effect of receiving prosocial contact on future allogrooming behavior” section. ∼24 hours later, the subject or the partner was placed in the center of an open field in a square chamber (50 cm × 50 cm × 50 cm) and allowed to freely explore the arena for 15 minutes. The location of the animal was tracked using the SLEAP software (https://github.com/talmolab/sleap/releases/tag/v1.3.3)^50^ and was then used to calculate the fraction of time spent in the corners (four 12.5 cm × 12.5 cm areas) and the total distance traveled.

##### Elevated plus maze

The elevated plus maze apparatus consisted of two open arms (30 cm × 5 cm) and two enclosed arms (30 cm × 5 cm) extending from a central intersection platform (5 cm × 5 cm). The apparatus was placed 75 cm above the floor. The animals first underwent phase 1 of the experiment described in the “assessment of the effect of receiving prosocial contact on future allogrooming behavior” section. ∼ 24 hours later, the subject or the partner was placed in the central chamber of the elevated plus maze and allowed to freely explore in the apparatus for 15 minutes. The location of the animal was tracked using the SLEAP software (https://github.com/talmolab/sleap/releases/tag/v1.3.3)^50^ and was then used to calculate the fraction of time spent in the closed arms.

##### Assessment of dominance status

Three days after the experiments in phase 2, the dominance relationship between the subject animal and the partner used in phase 2 was assessed using the tube test.^51^ The two animals were placed at opposite ends of a closed acrylic tube (length 60 cm; circumference 2.5 cm) and the first animal to retreat out of its end of the tube was designated as the loser and the other animal the winner. The assay was repeated five times and the animal that won at least three out of five times was considered dominant and the other animal subordinate. The animals were habituated to the tube for 2 days before the test.

#### Immunohistochemistry

Age-matched male C57BL/6J mice were co-housed in pairs for at least 4 weeks before the experiments. Within each pair, one animal was randomly assigned as the “subject” and the other as the “partner”. On the day of testing, the subject mouse was removed from the home cage and underwent acute restraint stress for 30 minutes. Following the stress period, the subject was reunited with the partner in the home cage, with a wire mesh divider placed in the middle of the cage during their interaction. In the “with-experience” group, the subject and the partner were placed on the same side of the divider, allowing the partner to engage in direct social contact, including allogrooming, toward the stressed subject. In the “without-experience” group, the two animals were placed on different sides of the divider, preventing direct physical contact. After 2.5 hours of interaction to allow Fos protein expression, the subject animal was sacrificed and perfused with 4% PFA. The brain was dissected out and fixed in 4% PFA for 2 hours at room temperature, rinsed with 1× PBS, then placed in 30% sucrose overnight at 4°C. Brains were then rinsed in PBS, embedded in OCT, and frozen at −80°C until sectioning.

60 μm coronal sections were cut on a Leica CM1950 cryostat. Free-floating sections were washed in PBS three times for 10 minutes each, followed by blocking in PBS containing 0.1% Tween 20 (PBST) and 5% normal donkey serum (NDS) for 30 minutes at room temperature. Sections were incubated overnight at 4 °C with primary antibody (rabbit anti-Fos; SYSY, catalog # 226008; 1:1000 dilution in PBST and 5% NDS). Sections were then washed with PBST five times and incubated overnight at 4°C with secondary antibody (donkey anti-rabbit IgG, Alexa Fluor^TM^ 488; ThermoFisher Scientific, catalog # A-21206; 1:1000 dilution in PBST and 5% NDS). Sections were subsequently washed with PBS four times and mounted onto Superfrost Plus slides and coverslipped with mounting medium (Fluoromount-G; SouthernBiotech, catalog # 0100-20). Images were acquired using a Leica DM6 B wide-field fluorescence microscope with a 10× objective. Fos-positive cells in different brain areas were detected using the ‘Cell detection’ function in the QuPath software (https://qupath.github.io).^52^ The results were visually checked to ensure accuracy. Brain areas were defined based on Paxinos and Franklin's The Mouse Brain in Stereotaxic Coordinates.^49^ For the PIL, sections with an AP coordinate of approximately −5.2 mm to −5.3 mm were included. For the ACC, sections with an AP coordinate of approximately 1.0 mm to 1.1 mm were included. For the MeA, sections with an AP coordinate of approximately −1.6 mm to −1.7 mm were included. For the MPOA, sections with an AP coordinate of approximately 0.0 mm to 0.1 mm were included. Results from sections in the same hemisphere were averaged.

### Quantification and statistical analysis

All statistical analyses were conducted using Prism (v10, GraphPad) or MATLAB (R2022b, MathWorks). Details about the types of statistical tests used, sample sizes, and other statistical information are provided in the figure legends and Table S1. The types of statistical tests were determined based on the experimental design and data distribution. P values were corrected for multiple comparisons when necessary. The center line in the boxplots indicates the median, the box limits indicate the upper and lower quartiles, and the whiskers indicate the range from the minimum to the maximum value. For analyses in which statistical significance was not observed in Figures 3, 4, 6, and S1–S4, power analyses were performed based on the observed effect sizes. These analyses indicate that statistical significance would still not be reached even with a sample size of 25 (with a statistical power of 0.9). Experiments were conducted using multiple batches of animals (with different dates of birth) to ensure that the findings are generalizable across cohorts. Animals of appropriate genotype, sex, age, and weight were randomly assigned to the experimental or control group. Test order was counterbalanced across animals whenever necessary. Experimenters were not blind to group allocation during data acquisition or analysis. All behaviors were annotated using pre-established criteria, and videos from the experimental and control groups were annotated side by side in an alternating manner to minimize temporal bias. All behavioral annotations were independently verified by a second annotator to enhance accuracy.

## Supplemental information

Document S1. Figures S1–S4

Table S1. Statistical information
