# Supplementary material for: Experience-dependent modulation of prosocial touch in mice
Source: iScience. 2026 Feb 20;29(3):115102. doi: 10.1016/j.isci.2026.115102 (PMC12992503; doi:10.1016/j.isci.2026.115102)
Supplement: Document S1. Figures S1–S4 [file mmc1.pdf]

iScience, Volume *29*

Supplemental information

Experience-dependent modulation of prosocial touch in mice

Yuhan Sun, Weizhe Hong, and Ye Emily Wu

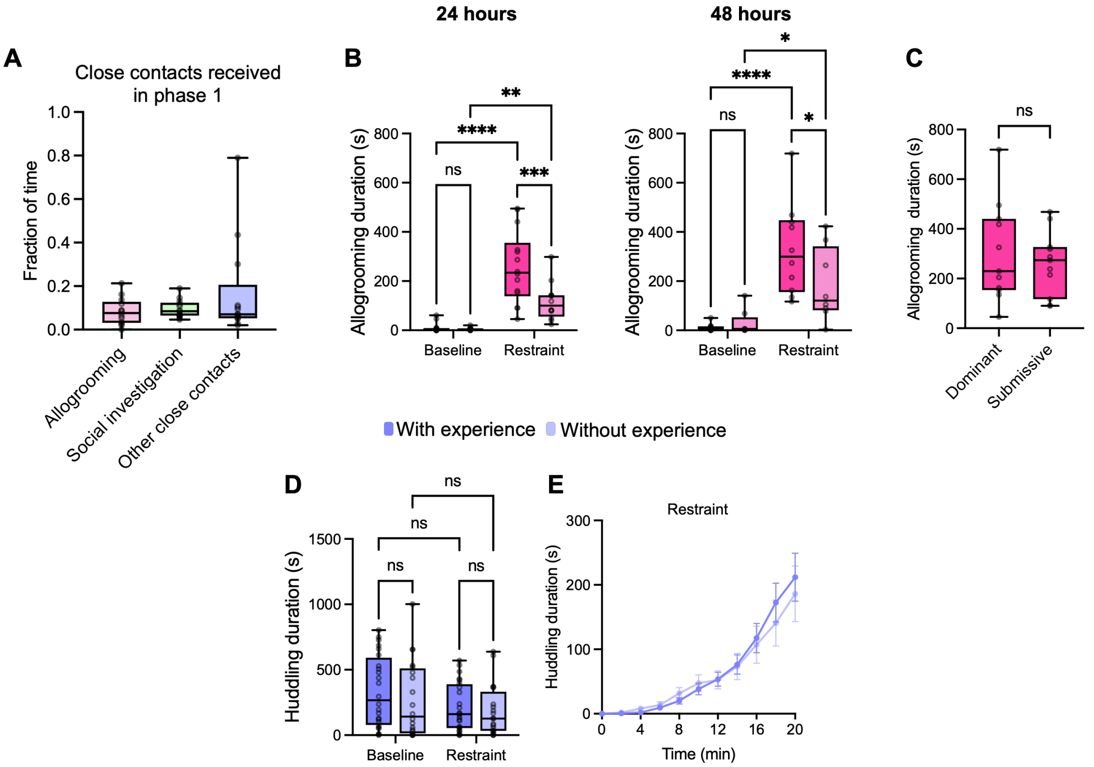

Figure S1. Male mice exhibit reciprocity in prosocial allogrooming, related to Figures 1 and 2.

(A) The fraction of time that male subject animals receive allogrooming, social investigation, and other forms of close contact (including those occurring during huddling or locomotion) in phase 1.

(B) The total duration of allogrooming displayed by male subject animals during interactions with unstressed or stressed partners at 24 hours (left) or 48 hours (right) after phase 1.

(C) The total duration of allogrooming displayed by dominant or submissive male subject animals in the “with experience” group toward stressed partners. The dominance relationship between the subject animal and the partner used in phase 2 was assessed using the tube test (Star Methods).

(D, E) The total duration (D) and cumulative duration (mean ± SEM) at various time points (E) of huddling behavior displayed by male subject animals during interactions with unstressed or stressed partners. Periods during which the subject animal engaged in allogrooming or investigation toward the partner while huddling were excluded.

n = 13 male mice in (A). n = 14 male mice in the “with experience” group and 12 male mice in the “without experience” group in (B, left). n = 10 male mice in the “with experience” group and 8 male mice in the “without experience” group in (B, right). n = 11 male mice in the dominant group and 11 male mice in the submissive group in (C). n = 24 male mice in the “with experience” group and 20 male mice in the “without experience” group in (D, E). The whiskers in the boxplots indicate the range from the minimum to the maximum value. (B) Two-way repeated measures analysis of variance (ANOVA) with post hoc Fisher's LSD test. (C), Two-sided Wilcoxon rank-sum test. (D), Two-way repeated measures analysis of variance (ANOVA) with post hoc Šidák multiple comparisons test. ****$p < 0.0001$. ***$p < 0.001$. **$p < 0.01$. *$p < 0.05$. ns, not significant. See Table S1 for additional statistical details.

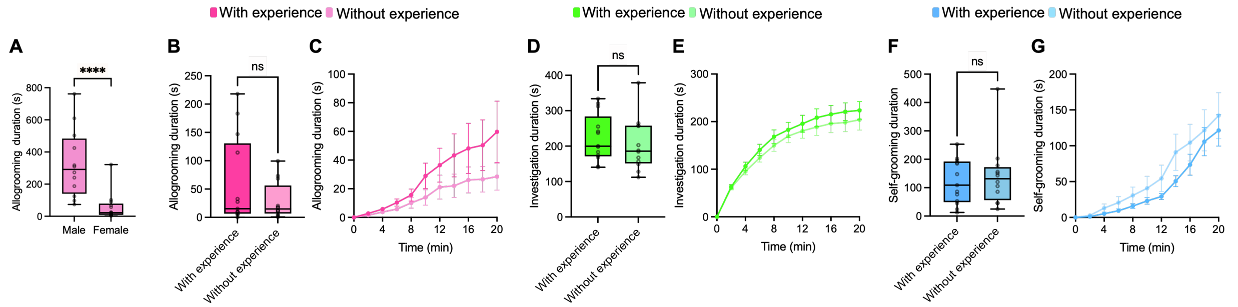

Figure S2. Examination of reciprocity in prosocial allogrooming in female mice, related to Figures 1 and 2.

(A) Comparison of the total duration of allogrooming received by male and female subjects during prior prosocial interactions in phase 1.

(B–G) The total duration (B, D, F) and cumulative duration (mean ± SEM) at various time points (C, E, G) of allogrooming (B, C), social investigation (D, E), and self-grooming (F, G) displayed by female subject animals during interactions with stressed partners in the “with experience” and “without experience” groups.

n = 12 male mice and 13 female mice in (A). n = 13 female mice in the “with experience” group and 13 female mice in the “without experience” group in (B–G). The whiskers in the boxplots indicate the range from the minimum to the maximum value. (A, B, D, F), Two-sided Wilcoxon rank-sum test. ****$p < 0.0001$. ns, not significant. See Table S1 for additional statistical details.

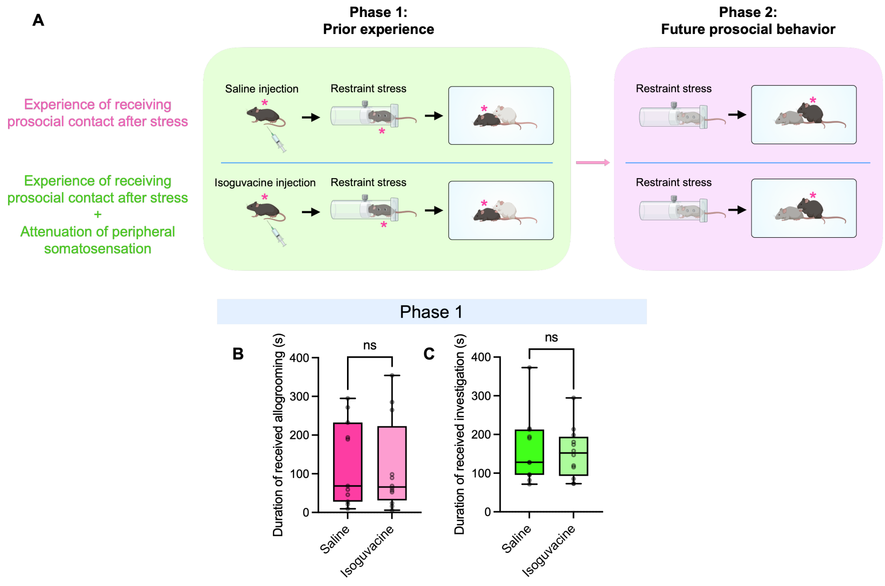

Figure S3. Attenuation of peripheral somatosensation does not affect the amount of allogrooming received, related to Figure 5.

(A) Schematic of the two-phase experimental paradigm for evaluating how attenuating peripheral somatosensation influences the effect of prior receipt of prosocial contact on subsequent allogrooming toward stressed partners.

(B, C) The total duration of allogrooming (B) and social investigation (C) received by stressed subjects (injected with either isoguvacine or saline) from their partners during the first 20 minutes of the interaction in the first experimental phase.

n = 12 male mice in the "isoguvacine" group and 11 male mice in the "saline" group. The whiskers in the boxplots indicate the range from the minimum to the maximum value. (B, C), Two-sided Wilcoxon rank-sum test. ns, not significant. See Table S1 for additional statistical details.

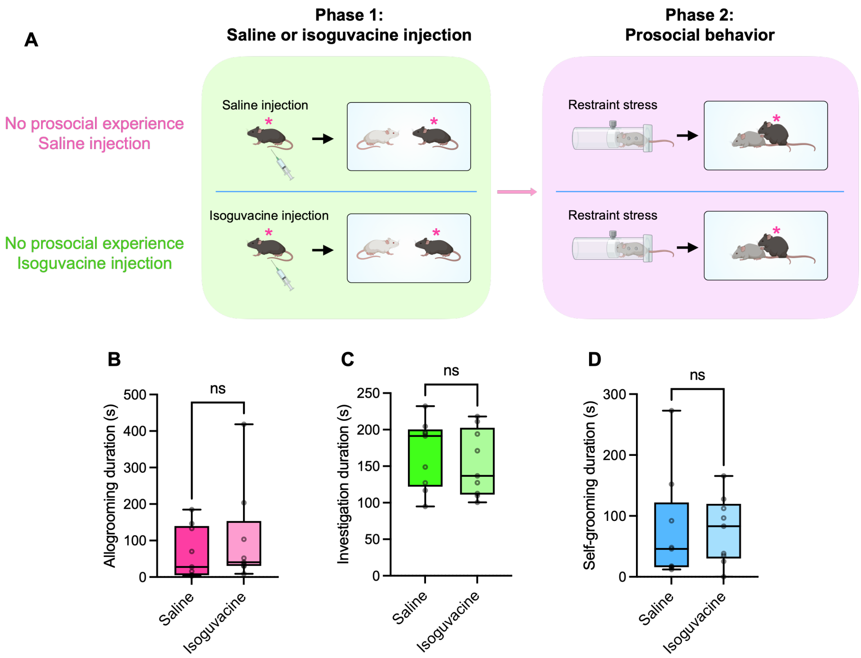

Figure S4. Administration of isoguvacine without prior prosocial interaction does not alter subsequent allogrooming behavior, related to Figure 5.

(A) Schematic of the two-phase experimental paradigm for evaluating how isoguvacine administration without prior prosocial interaction affects subsequent allogrooming toward stressed partners.

(B–D) The total duration of allogrooming (B), social investigation (C), and self-grooming (D) displayed by subject animals during interactions with stressed partners in the "isoguvacine" and "saline" groups.

n = 9 male mice in both the "isoguvacine" group and the "saline" group. The whiskers in the boxplots indicate the range from the minimum to the maximum value. (B–D), Two-sided Wilcoxon rank-sum test. ns, not significant. See Table S1 for additional statistical details.
